# Ultrafast hole carrier relaxation dynamics in p-type CuO nanowires

**DOI:** 10.1186/1556-276X-6-622

**Published:** 2011-12-07

**Authors:** Andreas Othonos, Matthew Zervos

**Affiliations:** 1 Research Center of Ultrafast Science, Department of Physics, University of Cyprus, PO Box 20537, Nicosia, 1678, Cyprus; 2 Nanostructured Materials and Devices Laboratory, Department of Mechanical Engineering, Materials Science Group, School of Engineering, University of Cyprus, PO Box 20537, Nicosia, 1678, Cyprus

**Keywords:** CuO nanowires, pump-probe spectroscopy, carrier dynamics

## Abstract

Ultrafast hole carrier relaxation dynamics in CuO nanowires have been investigated using transient absorption spectroscopy. Following femtosecond pulse excitation in a non-collinear pump-probe configuration, a combination of non-degenerate transmission and reflection measurements reveal initial ultrafast state filling dynamics independent of the probing photon energy. This behavior is attributed to the occupation of states by photo-generated carriers in the intrinsic hole region of the p-type CuO nanowires located near the top of the valence band. Intensity measurements indicate an upper fluence threshold of 40 μJ/cm^2 ^where carrier relaxation is mainly governed by the hole dynamics. The fast relaxation of the photo-generated carriers was determined to follow a double exponential decay with time constants of 0.4 ps and 2.1 ps. Furthermore, time-correlated single photon counting measurements provide evidence of three exponential relaxation channels on the nanosecond timescale.

## Introduction

Low-dimensional semiconductor nanostructures have been an active field of research due to their fascinating physical properties which are very different from their bulk counterparts. An important class of such nanostructures are semiconductor nanowires [NWs] since they may be used as building blocks for electronic and photonic devices which have attracted a great deal of attention over the past few years [1-3]. So far, a broad variety of metal-oxide [MO] NWs such as ZnO, SnO_2_, In_2_O_3_, and Ga_2_O_3 _have been grown and investigated in view of their potential technological applications. However, most of these MO NWs are n-type due to donors like oxygen-related defect states that reside energetically close to the conduction band edge. Moreover, p-type doping is not straightforward which limits the formation of p-n junctions and the realization of nanoscale electronic and optoelectronic devices. In contrast, cupric oxide [CuO] is a p-type semiconductor with a narrow bandgap energy (*E*_G_) reported to be between 1.3 and 1.7 eV [4]. The p-type conductivity is attributed to the presence of negatively charged copper vacancies and interstitial oxygen [5]. As such, CuO is regarded as an important material for applications in photovoltaic cells because of its high absorption coefficient, non-toxicity, and low cost.

Furthermore, CuO has been exploited as a heterogeneous catalyst to convert hydrocarbons into carbon dioxide and water [6]. However, while the growth of CuO NWs has been investigated and understood in some detail [7-9], it appears that very little work has been carried out on their optical properties while nothing is known about the ultrafast carrier dynamics despite their potential to be used as building blocks in optoelectronic devices.

In this work, CuO NWs were grown on Cu via a self-catalyzed vapor-solid mechanism using dry oxidation and subsequently transferred onto quartz and Si(001) substrates for femtosecond transient absorption spectroscopy measurements, providing a detailed understanding of the ultrafast carrier relaxation dynamics in this p-type semiconductor NWs. We find a fast negative change in absorption due to the occupation of states by the photo-generated carriers mainly in the intrinsic hole region near the top of the valence band and a recovery within a few picoseconds associated with redistribution of the photo-generated carriers in nearby energy states. Intensity measurements reveal an upper fluence threshold of 40 μJ/cm^2 ^where carrier relaxation is mainly governed by the hole dynamics. Furthermore, time-correlated single photon counting [TCSPC] photoluminescence [PL] measurements provide evidence of a three exponential relaxation dynamics on the nanosecond timescale.

## Experimental method

CuO NWs were grown using an atmospheric pressure chemical vapor deposition reactor which consists of four mass flow controllers and a horizontal quartz tube [QT] furnace capable of reaching a maximum temperature of 1,100°C. For the growth of the CuO NWs, we used high-purity Cu sheets with a thickness of ≈0.12 mm in the form of strips having a width of ≈3 mm and length of ≈10 mm. The Cu strips were flat and straight, were cleaned sequentially in methanol, acetone, and isopropanol [IPA] under ultrasonic vibration, and then rinsed in deionized [DI] H_2_O; after which, they were dipped in diluted hydrochloric acid at room temperature [RT] for 20 s in order to remove any oxides. Finally, the foil was rinsed thoroughly with DI H_2_O and dried with N_2_. Several strips were loaded into a porcelain-type boat which was positioned directly above the thermocouple used to measure the heater temperature at the center of the 1"; QT. The latter was flushed using an Ar-to-O_2 _ratio of 500:100 sccm for 10 min, and then the temperature was ramped to the growth temperature [*T*_G_] using a slow ramp rate of 5°C/min and 100 sccm of O_2_. Upon reaching *T*_G_, the same flow of 100 sccm O_2 _was maintained for a further 1 to 4 h. The furnace was then allowed to cool down to RT using the same flow of O_2_. The Cu foils turned black and were removed from the 1"; QT only when the temperature was < 100°C in order to prevent cracking due to stress. The morphology of the CuO NWs were then examined with a TESCAN scanning electron microscope [SEM] (TESCAN USA, Cranberry Twp., PA, USA) while their crystal structure and phase purity were investigated using a SHIMADZU, XRD-6000, X-ray diffractometer (Shimadzu Corporation, Nakagyo-ku, Kyoto, Japan) with a Cu-K*α *source, by performing a scan of *θ*-2*θ *in the range between 10° and 80°.

The dynamic behavior of carriers within the CuO NWs was investigated by measuring the temporal behavior of ultrafast time-resolved absorption obtained from simultaneous measurements of time-resolved transmission and reflection [10-12]. The experiments were carried out using a Ti/sapphire ultrafast amplifier system generating 100-fs pulses at 800 nm and running at a repetition rate of 1 kHz. A nonlinear crystal was used to generate 400 nm for the purpose of exciting the NWs, whereas part of the fundamental was used to generate a super continuum light for probing different energy states. Measurements were carried out using a typical pump-probe optical setup in a non-collinear configuration where differential reflection and transmission were utilized to determine the transient absorption.

## Results and discussion

**Figure 1:**
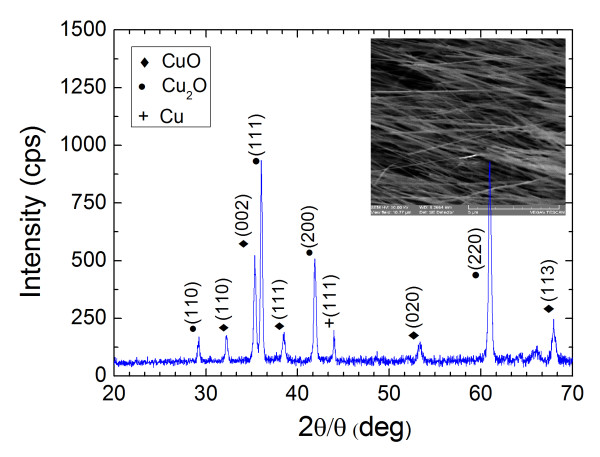
**XRD spectrum of the CuO NWs**. The CuO NWs were grown at 600°C for 4 h under 100 sccm of O_2 _identified by the (110), (002), (111), and (113) peaks. The inset shows an SEM image of the as-grown CuO NWs.

A high yield uniform distribution of CuO NWs with a characteristic black appearance was obtained on the thin Cu foil. A typical SEM image of the CuO NWs that were grown at 600°C for 1 h is shown as an inset in Figure 1. The CuO NWs are straight and have diameters ≤ 200 nm and lengths of up to 10 μm. These exhibited clear peaks in the XRD corresponding to the monoclinic crystal structure of CuO, while additional peaks were also observed due to the underlying red-like Cu_2_O and Cu foils. The CuO NWs grow in a self-catalyzed fashion via the formation of a strained layer of Cu_2_O on Cu and the subsequent diffusion of Cu and O_2 _through grain boundaries.

**Figure 2:**
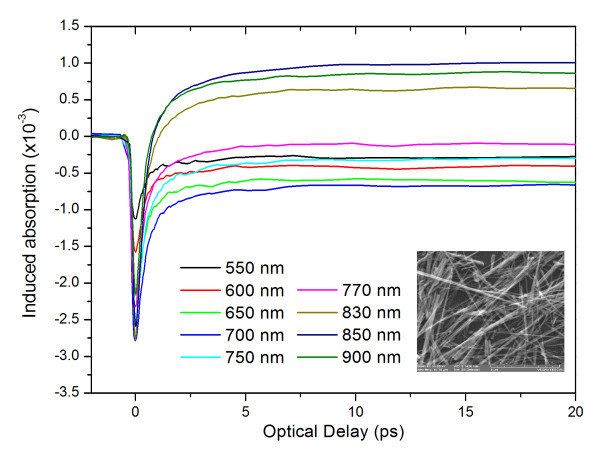
**CuO NWs' time-resolved differential absorption with 3.1-eV (≡400 nm) excitation and different wavelength probing**. The estimated absorbed fluence was 210 μJ/cm^2^. The lower inset shows the SEM image of the transferred wires.

As-grown samples were carefully immersed into a vial containing a few milliliters of IPA under ultrasonic vibration for short time intervals between 1 and 10 s in order to harvest CuO NWs and transfer them onto Si(001) or quartz for the purpose of optical spectroscopy. The light gray solution obtained after sonication was applied drop by drop on Si(001) and quartz, allowing the solvent to evaporate in between. A typical distribution of CuO NWs obtained in this way on Si(001) is shown as an inset in Figure 2.

Ultrafast, time-resolved, differential absorption measurements for the CuO NWs using different probing wavelengths are shown in Figure 2. The femtosecond excitation pulse was set at 3.1 eV with the absorbed fluence estimated to be 210 μJ/cm^2^. At first sight, the behavior appears to be similar for all probing wavelengths; that is, there seems to be a fast decrease (pulse width limited) in optical absorption reaching a minimum, followed by a fast recovery toward a plateau within 5 to 10 ps, and then returning back to its equilibrium value over several nanoseconds.

**Figure 3:**
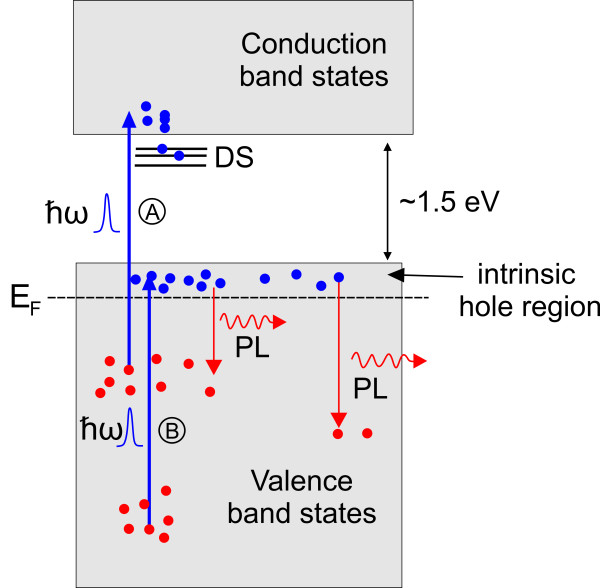
**A simplified schematic of the CuO band structure**. It is with an intrinsic hole population at the top of the valence band. Generation of non-equilibrium carrier densities via processes A and B are also shown in the diagram.

The observed transient absorption changes in a semiconductor following the above bandgap femtosecond pulse excitation are associated with the generation of non-equilibrium carrier density via the excitation of electrons from the valence to the conduction band (seen in Figure 3 as process A). However, in a p-type semiconductor like CuO, in addition to the normal excitation of electrons from the valence to the conduction band, the excitation of electrons within the valence band states, from the states below the Fermi energy to the available empty hole states (seen in Figure 3 as process B) near the top of the valence band [4], has an important contributing factor to the transient absorption changes. As the system evolves toward equilibrium, the photo-generated carriers distribute themselves along the energy states that are normally unoccupied under equilibrium conditions. In our measurements, this appears as a negative change in the induced absorption and is observed for all the probing photon energies (above and below the bandgap energy) with similar initial recovery. In view of this, along with the fact that we are probing a p-type semiconductor with a large intrinsic hole density [13], we believe that the observed initial drop in absorption is mainly associated with the occupation of states in the valence bands/sub-bands while its recovery corresponds to photo-generated carriers moving out of the probing region. On the other hand, the observed long-lived negative signal for probing energies larger than 1.5 eV corresponds to the contributions from additional state filling at different energy states, possibly due to the occupation of defect states[DS]/surface states (DS is seen in Figure 3), conduction band, or lower valence band states.

A competing process known as 'free-carrier absorption' is also likely to occur due to secondary excitation of the photo-generated carriers by the probing photons from their initial states to higher energy states. This phenomenon will result in a positive change in the induced absorption and is observed for probing wavelengths larger than 830 nm as seen in Figure 2, where carriers undergo secondary excitation. We should point out that the temporal behavior of the recovery which is on a nanosecond timescale is the same as the one observed for the long-lived state filling, suggesting that we are probing the same states. The observed long-lived behavior in comparison with the fast recovery of the initial state filling signifies the difference of the probing states. This suggests that for the long-lived behavior, we are probably probing defects/surface states rather than valence or conduction band states.

**Figure 4:**
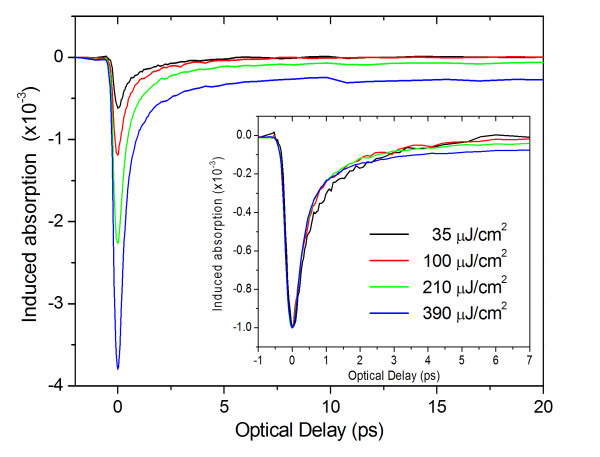
**CuO NWs' intensity time-resolved differential absorption with 3.1-eV excitation (400 nm) and 770-nm probing**.

Intensity measurements shown in Figure 4 reveal a linear increase in the peak of the induced absorption with the incident fluence reaching saturation at the 390 μJ/cm^2^. Here, we should point out that there is a slight decrease in the recovery with the increasing fluence. Similar results have been obtained for the other probing wavelengths. Intensity measurements also reveal that at fluence lower than 40 μJ/cm^2^, there is a negligible long-lived state filling contribution; therefore, the dynamics depict the temporal behavior of the top valence band holes. The best fit to these data was obtained with a double exponential function with time constants (associated strengths) of 0.4 ps (67%), associated with a redistribution of the photo-generated carrier in nearby energy states, and 2.1 ps (33%) corresponding to the carrier relaxation to further energy states from the probing region.

**Figure 5:**
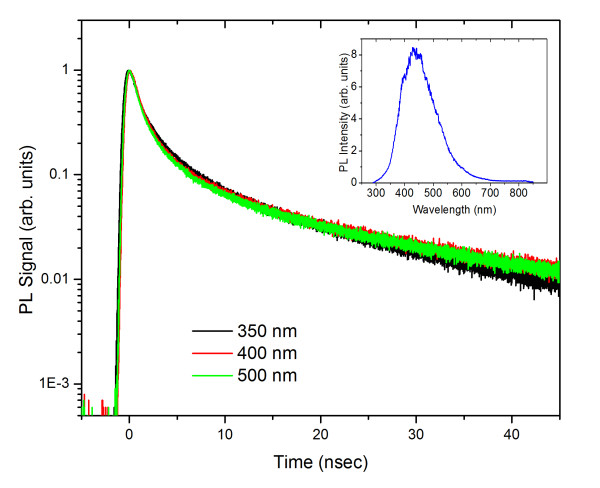
**CuO NWs' TCSPC PL excited with 267-nm ultrafast pulses and probe at 350, 400, 500 nm**. The inset shows a steady state PL spectrum from CuO NWs that were transferred on Si(001).

To obtain a better understanding of the dynamics in the CuO NWs on a longer time scale, time-resolved room temperature PL was carried out using TCSPC. The excitation was provided using a Ti/sapphire ultrafast amplifier, generating 267 nm at a repetition rate of 250 kHz. The spot size of the focused beam was approximately 2 mm. A further reduction of the incident intensity using a neutral density filter in front of the laser source revealed that the time constants were independent of the incident intensity. Figure 5 shows the TCSPC data obtained at several emission wavelengths namely at *λ *= 350, 400, and 500 nm covering the peak spectra region of the broad PL emission from the CuO NWs, as shown in the inset at the upper corner [14]. The same RT PL was obtained directly from the as-grown CuO NWs. We should point out that the PL emission from CuO as demonstrated from a previous work has drastically different profiles, depending on the sample preparation conditions. Given the narrow bandgap of CuO and the intrinsic hole concentration of this p-type semiconductor system, the PL emission following photoexcitation is most likely associated with the recombination of electrons from the top to the lower valence band states as well as the contribution from surface or defects states within the gap (Figure 3).

The decay profiles of the TCSPC measurements are approximately the same for the different probing wavelengths. The decays are well described by an exponential function of the form I*(t) *= A_1_*e^-t/τ^_1 _*+ A_2_*e^-t/τ^_2 _*+ A_3_*exp^-t/τ^_3_*, suggesting a complex energy level structure for the CuO NWs. The average exponential time constants and their associated strengths for the three probing wavelengths are 1.0 ns (80%), 4.8 ns (18%), and 26 ns (2%). This multi-exponential decay of the PL signal indicates the existence of non-radiative channels available to the probing carriers. Although the measured PL signal comes from a particular energy state, the non-radiative channels available to the photo-generated carriers in these states alter the population, thus making the PL decay appear multi-exponential.

## Conclusions

In conclusion, CuO NWs have been grown by dry oxidation of high-purity Cu foils at 600°C which were harvested and transferred on quartz and Si(001) substrates. Ultrafast transient absorption measurements reveal an initial pulse width-limited negative change in the induced absorption for all probing wavelengths associated with the occupation of photo-generated carriers of intrinsic hole states in the valence band of the p-type CuO NWs. The recovery of the initial state filling for the low incident fluence (< 40 μJ/cm^2^) suggests two relaxation mechanisms: a fast relaxation of 0.4 ps (67%) associated with redistribution of the photo-generated carriers around the probing region and a slower relaxation mechanism of 2.1 ps (33%) corresponding to a decay channel coupled to states further from the probing region. On a longer time scale, the TCSPC measurements show decay profiles which are approximately the same for the probing wavelengths between 350 and 500 nm. The data show multi-exponential decay with time constants at 1.0 ns (80%), 4.8 ns (18%), and 26 ns (2%).

## Abbreviations

IPA: isopropanol; MO: metal oxide; NWs: nanowires; PL: photoluminescence; SEM: scanning electron microscope; TCSPC: time-correlated single photon counting.

## Competing interests

The authors declare that they have no competing interests.

## Authors' contributions

The authors contributed equally, read, and approved the final manuscript.
